# Vitamin D status and seroconversion for COVID-19 in UK healthcare workers

**DOI:** 10.1183/13993003.04234-2020

**Published:** 2021-04-08

**Authors:** Aduragbemi A. Faniyi, Sebastian T. Lugg, Sian E. Faustini, Craig Webster, Joanne E. Duffy, Martin Hewison, Adrian Shields, Peter Nightingale, Alex G. Richter, David R. Thickett

**Affiliations:** 1 Birmingham Acute Care Research Group, Institute of Inflammation and Ageing, University of Birmingham, Birmingham, UK; 2 Clinical Immunology Service, Institute of Immunology and Immunotherapy, University of Birmingham, Birmingham, UK; 3 University Hospitals Birmingham NHS Foundation Trust, Birmingham, UK; 4 Institute of Metabolism and Systems Research, University of Birmingham, Birmingham, UK; 5 Joint first authors; 6 Joint last authors

## Abstract

The coronavirus disease 2019 (COVID-19) pandemic is a global health emergency, resulting in over 50 million infections and over 1.2 million deaths as of mid-November 2020 [1]. Healthcare workers are at a high risk of COVID-19 with large numbers of deaths reported around Europe and the UK, particularly among staff in the Black, Asian and minority ethnic (BAME) demographic group [2]. COVID-19 has disproportionately affected BAME individuals even after accounting for age, sex, social deprivation and comorbidity [3].

## Abstract 2

**NHS staff with vitamin D deficiency were more likely to have developed COVID-19, with staff from BAME ethnicity being the most vitamin D deficient** https://bit.ly/2J3kVTc

*To the Editor*:

The coronavirus disease 2019 (COVID-19) pandemic is a global health emergency, resulting in over 50 million infections and over 1.2 million deaths as of mid-November 2020 [1]. Healthcare workers are at a high risk of COVID-19 with large numbers of deaths reported around Europe and the UK, particularly among staff in the Black, Asian and minority ethnic (BAME) demographic group [2]. COVID-19 has disproportionately affected BAME individuals even after accounting for age, sex, social deprivation and comorbidity [3].

Vitamin D deficiency (VDD) is common in those of BAME ethnicity [4]. In VDD, both innate and adaptive immunity becomes dysregulated, increasing the risk of respiratory infection, as seen in influenza, common cold viruses and tuberculosis [5, 6]. There has been a great deal of interest in the role of VDD in COVID-19, with recent evidence suggesting VDD is more frequent in patients with severe COVID-19 compared to mild cases [7]. The prevalence of VDD and association with COVID-19 in healthcare workers has not been investigated.

We hypothesised that VDD was more common in healthcare workers who have seroconverted for COVID-19. This study defined the factors associated with VDD and the relationship between COVID-19 seroconversion and VDD.

This cross-sectional observational study recruited healthcare workers between 12 and 22 May, 2020 from the University Hospitals Birmingham NHS Foundation Trust (UHBFT) across four sites. This was a sub-study of the COVID-19 convalescent immunity study (COCO) approved by the London – Camden and Kings Cross Research Ethics Committee (20/HRA/1817). The main inclusion criteria were staff members who had isolated for symptoms suggestive of COVID-19. After obtaining consent, blood samples were taken for measurement of vitamin D levels by mass spectrometry, and anti-SARS-CoV-2 spike glycoprotein antibodies using a combined IgG, IgA, IgM ELISA (The Binding Site; product code: MK654) [8]. This CE marked assay has 98.6% (95% CI 92.6–100) sensitivity and 98.3% (95% CI 96.4–99.4) specificity. The median time from symptom onset to sample collection was 48 days. COVID-19 seroconversion was used to indicate previous infection with severe acute respiratory syndrome coronavirus 2 (SARS-CoV-2). VDD was defined as serum 25(OH)D_3_ concentration <30 nmol·L^−1^ as per the UHBFT clinical laboratory reference range and the UK National Osteoporosis Society guidance [9].

Of the 392 healthcare workers studied, 61 (15.6%) had VDD. The participant demographics, occupation and seroconversion status are displayed in table 1. Those with VDD were significantly more likely to be BAME (p<0.0001) and in a junior doctor job role (p=0.029); there were no differences in age, body mass index or comorbidity status between those with VDD and those without. Using backwards logistic regression to determine factors associated with VDD, the multivariate analysis used all demographic variables in table 1. The significant independent factors for VDD were BAME (OR 8·86, 95% CI 4.75–16.52; p<0.001) and seroconversion (OR 2.15, 95% CI 1.11–4.17; p=0.023). The overall predictive power of the model was 77.9% (95% CI 71.1–84.7, se 3.5%; p<0.001) indicated by the area under the receiver operator characteristic (ROC) curve.

**TABLE 1:** Participant demographic, occupation and seroconversion status

	**Total**	**Vitamin D deficient**	**Non-vitamin D deficient**	**p-value**
**Subjects n**	392	61	331	
**Age years**	41 (30–50)	35 (28–47.5)	42 (31–50)	0.073
**Gender**				
Female	285 (73%)	40 (66%)	245 (74%)	0.112
Male	100 (26%)	21 (34%)	79 (24%)	
Not stated	7 (2%)		7 (2%)	
**BMI kg·m^−2^**	25.9 (22.9–30.1)	25.4 (22.9–30.8)	26.0 (22.9–30.1)	0.794
**Ethnicity**				
White	279 (71%)	18 (30%)	261 (79%)	<0.0001
BAME	108 (28%)	43 (70%)	65 (20%)	
Not stated	5 (1%)		5 (2%)	
**Comorbidities**				
None	240 (61%)	40 (66%)	200 (60%)	0.478
One or more	152 (39%)	21 (34%)	131 (40%)	
**Job role**				
Junior doctor	50 (13%)	15 (25%)	35 (11%)	0.029^#^
Consultant	65 (17%)	7 (11%)	58 (18%)	
Junior nurse	65 (17%)	12 (20%)	53 (16%)	
Senior nurse	66 (17%)	7 (11%)	59 (18%)	
Physiotherapist	28 (7%)	4 (7%)	24 (7%)	
Laboratory worker	26 (7%)	7 (11%)	19 (6%)	
Radiology/theatre staff/ pharmacy	21 (5%)	1 (2%)	20 (6%)	
Secretary/administrator	35 (9%)	2 (3%)	33 (10%)	
Healthcare assistant/ phlebotomist	36 (9%)	6 (10%)	30 (9%)	
**Seroconversion**				
Yes	214 (55%)	44 (72%)	170 (51%)	0.003
No	178 (45%)	17 (28%)	161 (49%)	

Data are presented as median (interquartile range) or n (%), unless otherwise indicated. Vitamin D deficient is Serum 25(OH)D_3_ <30 nmol·L^−1^, while not deficient is ≥30 nmol·L^−1^. Where proportions are shown, they were calculated using the n numbers shown in columns as denominator; p-values were calculated using Mann–Whitney test for data presented as median and interquartile range, and by Fisher's exact test for data presented as n (%), with p<0.05 considered significant. The sample size for the backward logistic regression is 379, with 13 excluded from this analysis; seven because gender was not stated, five due to ethnicity not stated and one with body mass index (BMI) unknown. Age and BMI were treated as continuous variables, job role as nine categories, while all others were treated as dichotomous. Eight participants were taking vitamin D supplements, one in the vitamin D deficient group and seven in the non-vitamin D deficient group. Comorbidities were classified as shown because they were uncommon in this cohort, with the most common comorbidities being hypertension (n=34, 9%) and asthma (n=26, 7%). ^#^: p-value of 0.226 when excluding junior doctor group in analysis. BAME: Black, Asian and minority ethnic demographic group.

Self-reported symptoms in healthcare workers (n=386), included fever (n=235, 61%), breathlessness (n=186, 48%), cough (n=117, 30%), loss of smell/taste (n=169, 44%), body aches/pains (n=274, 71%), fatigue (n=339, 88%), diarrhoea (n=115, 30%) and sore throat (n=197, 51%). VDD staff experienced more body aches/pains (82% *versus* 69%; p=0.045) but there were no significant differences in other symptoms reported between groups.

Seroconversion was higher in healthcare workers with VDD compared to those without (n=44/61, 72% *versus* n=170/331, 51%; p=0.003), representing an absolute increase of 13 cases in the VDD group. Seroconversion was higher in BAME males with VDD compared to those without (n=17/18, 94% *versus* n=12/23, 52%; p=0.005); no differences were observed in the other ethnic–gender sub-analysis. Using backwards logistic regression to determine factors associated with seroconversion, the multivariate analysis used all the variables from table 1; only VDD was a significant independent risk factor for developing seroconversion (OR 2.6, 95% CI 1.41–4.80; p=0.002). The overall predictive power of the model was 55·5% (95% CI 49.8–61.2, se 2.9%; p=0.06) as indicated by the area under the ROC curve.

To our knowledge this is the first study to investigate VDD prevalence in a UK healthcare worker cohort. VDD was relatively uncommon (15.6%) and lower than healthcare worker studies published in the USA and Gulf Areas, which may in part reflect differences in reference ranges and vitamin D assays used [10]. The increase in VDD seen in junior doctors echoes previous findings of junior doctors have lower levels than senior doctors [10], which may be due to increased frontline work and different shift patterns.

Our data supports previous findings of higher VDD in BAME ethnicity [4]. While BAME was not an independent risk factor for seroconversion, our sub-group analysis found that VDD BAME males may be most at risk from COVID-19 as there was remarkably high seroconversion rate of 94% in this sub-group. Although this is a cohort of mild COVID-19, being BAME and male are known risk factors for a severe outcome from COVID-19.

Our study showing VDD as an independent risk factor for COVID-19 seroconversion is consistent with others including a large US study which found that COVID-19 positivity was inversely related to patient vitamin D levels in the preceding 12 months [11]. Additional data from Israel found low vitamin D increased the risk of COVID-19 positivity and COVID-19 related hospitalisation [12]. Furthermore, a recent open labelled clinical trial from Spain provided a proof of concept that high-dose vitamin D treatment may be useful therapy for severe COVID-19 [13].

The role of vitamin D in modulating the immune response to COVID-19 is likely to be multifactorial. Vitamin D increases the production of antimicrobial peptides in the respiratory epithelium, which may protect against viral infection. Also, vitamin D supplementation has been shown to reduce viral upper respiratory tract infections in metanalysis [14]. Vitamin D may help reduce the body's response to COVID-19; older patients with VDD (<30 nmol·L^−1^) had higher D-dimer levels, a marker of inflammation and vascular damage, and required more noninvasive ventilation support [15]. There is a bidirectional association between VDD and COVID-19 in our study and therefore it is unclear if VDD is a cause and/or consequence of COVID-19.

This study has several limitations. Firstly, staff were recruited from a single NHS trust, although it is the second largest in the UK, spanning four hospital sites. Secondly, as the healthcare workers had mild COVID-19, this study does not inform of the role of VDD in severe COVID-19. Thirdly, due to relatively small sample size, we were unable to analyse the differences between staff from different BAME sub-groups. Fourthly, there is a risk of bias from recruiting individuals with self-reported symptoms compared to a cross-sectional survey of all healthcare workers. Finally, other confounders such as sociocultural factors which may affect risk of transmission and/or VDD were not addressed.

In summary, in healthcare workers who have isolated due to symptoms of COVID-19, those of BAME ethnicity are at the highest risk of VDD. Furthermore, VDD was an independent risk factor for development of COVID-19 seroconversion, with the biggest differences seen in the BAME male group. Therefore, we suggest future high-dose vitamin D treatment trials should target such at risk groups within healthcare workers with aim of potentially preventing or alleviating COVID-19.

## Shareable PDF

10.1183/13993003.04234-2020.Shareable1 This one-page PDF can be shared freely online.
Shareable PDF ERJ-04234-2020.Shareable
