# Supplementary material for: Vitamin D status and seroconversion for COVID-19 in UK healthcare workers
Source: Eur Respir J. 2021 Apr 8;57(4):2004234. doi: 10.1183/13993003.04234-2020 (PMC7736751; doi:10.1183/13993003.04234-2020)
Supplement: Supplementary file 1 [file ERJ-04234-2020.Shareable.pdf]

AGORA
RESEARCH LETTER

SHAREABLE PDF

Vitamin D status and seroconversion for COVID-19 in UK healthcare workers

Aduragbemi A. Faniyi[1,5], Sebastian T. Lugg[1,5], Sian E. Faustini[2], Craig Webster[3], Joanne E. Duffy[3], Martin Hewison[4], Adrian Shields [2,3], Peter Nightingale[3], Alex G. Richter[2,3,6] and David R. Thickett[1,3,6]

Affiliations: [1]Birmingham Acute Care Research Group, Institute of Inflammation and Ageing, University of Birmingham, Birmingham, UK. [2]Clinical Immunology Service, Institute of Immunology and Immunotherapy, University of Birmingham, Birmingham, UK. [3]University Hospitals Birmingham NHS Foundation Trust, Birmingham, UK. [4]Institute of Metabolism and Systems Research, University of Birmingham, Birmingham, UK. [5]Joint first authors. [6]Joint last authors.

Correspondence: David R. Thickett, Institute of Inflammation and Ageing, University of Birmingham, Birmingham B15 2TH, UK. E-mail: d.thickett@bham.ac.uk

@ERSpublications
NHS staff with vitamin D deficiency were more likely to have developed COVID-19, with staff from BAME ethnicity being the most vitamin D deficient https://bit.ly/2J3kVTc

Cite this article as: Faniyi AA, Lugg ST, Faustini SE, *et al*. Vitamin D status and seroconversion for COVID-19 in UK healthcare workers. *Eur Respir J* 2021; 57: 2004234 [https://doi.org/10.1183/13993003.04234-2020].

This single-page version can be shared freely online.

To the Editor:

The coronavirus disease 2019 (COVID-19) pandemic is a global health emergency, resulting in over 50 million infections and over 1.2 million deaths as of mid-November 2020 [1]. Healthcare workers are at a high risk of COVID-19 with large numbers of deaths reported around Europe and the UK, particularly among staff in the Black, Asian and minority ethnic (BAME) demographic group [2]. COVID-19 has disproportionately affected BAME individuals even after accounting for age, sex, social deprivation and comorbidity [3].

Copyright ©ERS 2021.. This version is distributed under the terms of the Creative Commons Attribution Licence 4.0.
